# Survival analysis of 5595 head and neck cancers--results of conventional treatment in a high-risk population.

**DOI:** 10.1038/bjc.1998.249

**Published:** 1998-05

**Authors:** D. N. Rao, P. D. Shroff, G. Chattopadhyay, K. A. Dinshaw

**Affiliations:** Division of Epidemiology and Biostatistics, Tata Memorial Hospital, Parel, Mumbai, India.

## Abstract

This is a study of 5595 head and neck cancer patients treated during 1987-89 at TMH, Mumbai. The study included 1970 oral cancers (ICD 140-145), 1495 oropharyngeal cancers (ICD 1410, 1453, 146), 1255 hypopharyngeal cancers (ICD 148), 125 nasopharyngeal cancers (ICD 147) and 750 laryngeal cancers (ICD 161). The clinical extent of disease at presentation was based on TNM group staging (UICC 1978). For the majority of sites, patients attended the hospital during stage III and stage IV of the disease; the only exception was for cancers of the lower lip, anterior tongue and vocal cord when between 46.2% and 56.5% of patients with localized cancer (stage I and II) were seen. Generally, surgery either alone or with radiation has been administered for oral cancer patients whereas radiation either alone or in combination with chemotherapy was administered for other head and neck sites. The overall 5-year survival rate was in the range of 20-43% for oral cancer, 8-25% for pharyngeal cancers and 25-62% for laryngeal cancer. The 5-year relative survival rates were more or less in agreement with the results published by the Eurocare study for head and neck cancers. The importance of primary prevention in head and neck cancer is stressed.


British Journal of Cancer (1998) 77(9), 1514–1518
© 1998 Cancer Research Campaign

### Survival analysis of 5595 head and neck cancers – results of conventional treatment in a high-risk population

**DN Rao[1], PD Shroff[1], G Chattopadhyay[1] and KA Dinshaw[2]**

[1]Division of Epidemiology and Biostatistics and [2]Tata Memorial Center, Tata Memorial Hospital, Parel, Mumbai 400 012, India

**Summary** This is a study of 5595 head and neck cancer patients treated during 1987–89 at TMH, Mumbai. The study included 1970 oral cancers (ICD 140–145), 1495 oropharyngeal cancers (ICD 1410, 1453, 146), 1255 hypopharyngeal cancers (ICD 148), 125 nasopharyngeal cancers (ICD 147) and 750 laryngeal cancers (ICD 161). The clinical extent of disease at presentation was based on TNM group staging (UICC 1978). For the majority of sites, patients attended the hospital during stage III and stage IV of the disease; the only exception was for cancers of the lower lip, anterior tongue and vocal cord when between 46.2% and 56.5% of patients with localized cancer (stage I and II) were seen. Generally, surgery either alone or with radiation has been administered for oral cancer patients whereas radiation either alone or in combination with chemotherapy was administered for other head and neck sites. The overall 5-year survival rate was in the range of 20–43% for oral cancer, 8–25% for pharyngeal cancers and 25–62% for laryngeal cancer. The 5-year relative survival rates were more or less in agreement with the results published by the Eurocare study for head and neck cancers. The importance of primary prevention in head and neck cancer is stressed.

**Keywords:** head and neck cancer; survival; TNM; stage; treatment

Incidence data that are available from six metropolitan cities and one rural registry in India indicate that head and neck cancer is a common problem there (IARC, 1992). Many epidemiological studies carried out in the sub-continent have shown the association of tobacco, alcohol and some dietary items with head and neck cancer. Although primary prevention may be the ideal choice for the control of head and neck cancer, secondary prevention through therapeutic intervention has an equal and important role to play. Management of head and neck cancer in a high-risk population and its response to conventional treatment and survival have not been reported in detail. The aim is to analyse individual sites of head and neck cancer according to stage of the disease, primary treatment and other prognostic factors for 5-year survival. Comparison is also made with survival in European countries.

### PATIENTS AND METHODS

This is a retrospective analysis of 5595 eligible head and neck cancer patients who were diagnosed and treated at Tata Memorial Hospital, Mumbai, during the period 1987–89. The eligibility criteria for inclusion of patients in the study were: (1) no prior cancer-directed treatment at the time of registration; (2) histologically confirmed epithelial cancer; (3) treatment with chemotherapy together with surgery or radiation but not as the only treatment; and (4) at least 50 cases in each subsite of head and neck cancer. The excluded subsites were upper lip (five cases), commissure of lip (six cases), tongue NOS (not otherwise specified) (one case), salivary gland (39 cases), palate NOS (two cases) and subglottic larynx (seven cases). Information on age, sex, date of diagnosis, method of diagnosis, primary site (ICD 1978), secondary site, if available, histology of primary and/or secondary tumour, TNM staging (UICC, 1978) and primary treatment given within 6 months of diagnosis were obtained from the database maintained by the hospital cancer registry. The clinical extent of the disease for head and neck cancer cases was classified into four stages, viz. stage I comprising $T_1N_0M_0$ status, stage II comprising $T_2N_0M_0$, stage III comprising $T_3N_0M_0$, $T_3N_1M_0$, $T_1N_1M_0$ and $T_2N_1M_0$ and stage IV comprising $T_4N_0M_0$, $T_4N_1M_0$, and any T, $N_2$ or $N_3$ $M_0$ and any T any N $M_1$. Periodic updating of follow-up information was carried out either by scrutiny of medical records of attending patients or by postal enquiry responses. In some cases, follow-up information was also obtained by scrutiny of death records maintained by the Municipal Corporation of the City, of life insurance claims and of records from terminal care centres/pain clinic in the city. The study was closed on 31 March 1996, and data available up to that time were used for the survival analyses. In this study of 5595 patients, 2435 patients (43%) were known to have died and 1128 patients (20%) were known to be alive at the end of 5 years from their date of diagnosis. For the remaining 2032 patients, 267 patients (4.7%) had follow-up information for between 3 and 5 years, 1384 patients (24.7%), categorized as non-responders, had less than 3 years of follow-up information and 381 patients (6.8%) had incomplete addresses (untraced cases). Complete follow-up information was 68% for the laryngeal group, 65% for oral cancer, 64% for the oropharyngeal and nasopharyngeal group and 60% for the hypopharyngeal group. At the end of 1 year, the equivalent figures were about 84% for laryngeal cancer and 70–80% for the other groups.

*Received 5 August 1997*
*Revised 23 October 1997*
*Accepted 27 October 1997*

*Correspondence to:* DN Rao

**Table 1** Clinical characteristics and observed survival rates (%) for oral cancers 1987–89

| Site (ICD 9) | Lower lip (1401) | Ant. tongue (1411–14) | L. alvelous (1431) | U. alvelous (1430) | Floor mouth (1449) | Buccal mucosa (1450–51) | Hard palate (1452) | Retromolar (1456) |
|---|---|---|---|---|---|---|---|---|
| Number of cases | 62 | 522 | 340 | 71 | 88 | 728 | 90 | 69 |
| Sex ratio | 3.1:1 | 2.3:1 | 2.3:1 | 1.7:1 | 7.8:1 | 2.2:1 | 3.3:1 | 2.8:1 |
| Average age ± s.d. (years) | 50.7 ± 13.6 | 49.4 ± 11.9 | 52.2 ± 10.5 | 52.7 ± 13.6 | 51.1 ± 9.4 | 50.8 ± 11.7 | 54.1 ± 12.5 | 53.2 ± 9.2 |
| Stage (%) | | | | | | | | |
| I | 22.6 | 20.9 | 1.2 | 2.8 | 5.7 | 6.2 | 1.1 | 1.5 |
| II | 32.3 | 25.3 | 6.7 | 19.7 | 18.2 | 20.0 | 26.6 | 13.0 |
| III | 14.5 | 31.0 | 9.1 | 47.9 | 13.6 | 19.5 | 36.6 | 30.4 |
| IV | 29.0 | 21.3 | 81.2 | 22.5 | 58.0 | 52.1 | 17.9 | 53.6 |
| NOS | 1.6 | 1.5 | 1.8 | 7.1 | 4.5 | 2.2 | 17.8 | 1.5 |
| Treatment summary (%) | | | | | | | | |
| Surgery | 83.9 | 50.4 | 48.8 | 32.4 | 23.9 | 42.9 | 18.9 | 23.2 |
| Radiation | 1.6 | 18.8 | 6.5 | 21.1 | 36.4 | 19.1 | 56.7 | 39.1 |
| Surgery + radiation | 12.9 | 22.4 | 35.3 | 39.4 | 28.4 | 28.7 | 20.0 | 24.6 |
| Others[a] | 1.6 | 8.4 | 9.4 | 7.1 | 11.3 | 9.3 | 4.4 | 13.1 |
| Survival with CI | | | | | | | | |
| 1 year | 61 (49–73) | 55 (51–59) | 62 (57–68) | 46 (35–58) | 47 (36–57) | 61 (57–64) | 51 (41–61) | 56 (45–68) |
| 3 years | 48 (36–60) | 36 (32–40) | 35 (30–40) | 21 (12–31) | 23 (14–31) | 39 (35–42) | 34 (25–44) | 29 (18–40) |
| 5 years | 43 (31–56) | 33 (29–37) | 31 (26–36) | 20 (10–29) | 21 (13–30) | 34 (31–37) | 31 (21–40) | 25 (14–35) |
| Median survival (in months) | 33 | 17 | 19 | 12 | 12 | 20 | 16 | 17 |

[a]Includes chemotherapy either with surgery or with radiation or with both. CI, 95% confidence interval; NOS, not otherwise specified.

**Table 2** Clinical characteristics and observed survival rates (%) for pharyngeal and laryngeal cancers 1987–89

| Site (ICD 9) | Base tongue (1410) | Soft palate (1453) | Tonsil (1460) | Oropharynx NOS (1461–69) | Post cricoid (1480) | Pyriform fossa (1481) | Hypopharynx NOS (1482–89) | Vocal cord (1610) | Supra glottic (1611) | Nasopharynx (1470–79) |
|---|---|---|---|---|---|---|---|---|---|---|
| Number of cases | 818 | 142 | 346 | 189 | 171 | 1000 | 84 | 259 | 491 | 125 |
| Sex ratio | 11.4:1 | 7.4:1 | 8.6:1 | 6.3:1 | 0.9:1 | 12.3:1 | 3.4:1 | 17.5:1 | 7.8:1 | 4.2:1 |
| Average age ± sd (years) | 55.0±10.6 | 56.2±10.5 | 55.9±10.9 | 56.6±10.5 | 49.9±13.9 | 55.5±10.4 | 53.6±13.5 | 56.0±11.2 | 55.2±10.5 | 38.2±18.2 |
| Stage distribution (%) | | | | | | | | | | |
| I | 0.7 | 6.3 | 2.0 | 2.1 | 1.2 | 0.1 | 1.2 | 51.7 | 1.8 | 0.8 |
| II | 10.1 | 25.4 | 8.1 | 13.2 | 15.2 | 8.7 | 11.9 | 15.4 | 11.4 | 2.4 |
| III | 46.2 | 38.7 | 50.6 | 55.0 | 50.9 | 52.9 | 60.7 | 20.1 | 56.0 | 15.2 |
| IV | 41.7 | 27.5 | 37.3 | 28.0 | 29.8 | 37.0 | 25.0 | 9.7 | 25.7 | 76.8 |
| NOS | 1.3 | 2.1 | 2.0 | 1.7 | 2.9 | 1.3 | 1.2 | 3.1 | 5.1 | 4.8 |
| Treatment summary (%) | | | | | | | | | | |
| Surgery | 0.7 | 2.1 | 0.3 | 0 | 10.5 | 3.4 | 2.4 | 11.2 | 2.9 | 0 |
| Radiation | 86.2 | 81.7 | 87.3 | 90 | 74.3 | 79.1 | 84.5 | 74.9 | 85.7 | 50.4 |
| Surgery + radiation | 3.2 | 6.3 | 2.9 | 2.1 | 11.1 | 12.9 | 4.8 | 13.1 | 8.6 | 4.8 |
| Others[a] | 9.9 | 9.9 | 9.5 | 7.9 | 4.1 | 4.6 | 8.3 | 0.8 | 2.8 | 44.8 |
| Survival with CI | | | | | | | | | | |
| 1 year | 43 (39–46) | 58 (49–66) | 44 (39–49) | 45 (38–52) | 34 (26–40) | 48 (44–51) | 39 (29–50) | 82 (77–87) | 54 (41–58) | 50 (42–59) |
| 3 years | 19 (16–21) | 33 (25–41) | 15 (11–19) | 20 (14–26) | 16 (11–22) | 22 (19–24) | 13 (6–20) | 65 (59–71) | 28 (24–32) | 27 (19–35) |
| 5 years | 15 (12–17) | 25 (17–32) | 13 (9–16) | 14 (8–19) | 13 (8–18) | 17 (15–20) | 8 (2–14) | 62 (56–68) | 25 (21–29) | 21 (14–28) |
| Median survival (in months) | 10 | 18 | 11 | 11 | 7 | 12 | 9 | * | 14 | 13 |

[a]Includes chemotherapy either with surgery or with radiation or with both. [b]Not reached. CI, 95% confidence interval; NOS, not otherwise specified.

The number of untraced cases was around 7% for all sites, except laryngeal cancer for which the figure was 3.7%; similarly, the proportion of non-responders was around 25% for all sites except for laryngeal cancer (21.6%). The proportion of both untraced and non-responding patients who had stage III and stage IV cancers at their first visit to hospital was between 70% and 92% in the different site groups, and these patients were considered unsuitable for further treatment, except for pain relief or symptomatic treatment. Patients with less than 3 years of follow-up were considered as being deceased, and survival information available up to that time was used for analysis purposes. The Kaplan–Meier method was used to estimate survival rates for 1, 3 and 5 years and also to assess certain prognostic factors considered in the study. As 70% of the patients attended hospital from Maharashtra state, the life table for Maharashtra State, published by the Government of India for the period 1986–90 (RGI, 1994), was used to estimate the relative survival rates.

© *Cancer Research Campaign 1998*

**Table 3** Five-year observed survival rates (in %) for oral cavity, pharyngeal and laryngeal cancers by treatment

| Site | Surgery | Radiation | Surgery + radiation | Surgery + chemotherapy | Radiation + chemotherapy | Surgery + radiation + chemotherapy |
|---|---|---|---|---|---|---|
| Oral cavity | | | | | | |
| Lower lip | 47.8 | 0[a] | 25 | – | – | 0[a] |
| Anterior tongue | 47.8 | 13.2 | 22.0 | 33.3 | 11.1 | 27.2 |
| Lower alveolus | 34.4 | 13.6 | 28.9 | 45.0 | 20.0 | 14.3 |
| Upper alveolus | 30.4 | 13.3 | 17.1 | – | 0[a] | 0[a] |
| Floor mouth | 42.9 | 3.1 | 36.0 | – | – | 0[a] |
| Buccal mucosa | 46.4 | 23.3 | 27.9 | 30.7 | 6.7 | 26.3 |
| Hard palate | 58.8 | 19.6 | 38.9 | – | 25.0 | – |
| Retromolar | 37.5 | 14.8 | 41.2 | 0[a] | 0[a] | 0[a] |
| Oropharynx | | | | | | |
| Base tongue | 66.7 | 15.3 | 19.2 | 0[a] | 6.0 | 0[a] |
| Soft palate | 66.7 | 24.2 | 55.6 | – | – | 0[a] |
| Tonsil | 0[a] | 13.9 | 20.0 | – | 3.3 | 0[a] |
| Oropharynx NOS | – | 14.0 | 0[a] | – | 7.1 | 100[a] |
| Hypopharynx | | | | | | |
| Post-cricoid | 11.1 | 14.8 | 7.9 | – | 0 | 0[a] |
| Pyriform fossa | 14.1 | 15.6 | 32.5 | 0[a] | 7 | 100[a] |
| Hypopharynx NOS | 0[a] | 8.5 | 0[a] | – | – | – |
| Nasopharynx | – | 12.7 | 44.4 | – | 28.5 | 0[a] |
| Larynx | | | | | | |
| Vocal cord | 72.4 | 62.1 | 51.7 | – | 50.0 | – |
| Supraglottic | 28.6 | 25.2 | 48.0 | 0[a] | 25.0 | 0[a] |

[a]Estimate based on five or less cases. –, No patient.

**Table 4** Five-year relative survival rates (%) according to sex and TNM group staging for head and neck cancers 1987–89

| | Five-year relative survival (%) | | | | | |
|---|---|---|---|---|---|---|
| | Sex | | Stage | | | |
| | M | F | I | II | III | IV |
| Oral Cavity | | | | | | |
| Lower lip | 51.3 | 35.6 | 69.8 | 54.0 | 60.3 | 12.7 |
| Anterior tongue | 35.5 | 35.8 | 63.6 | 49.8 | 22.8 | 11.2 |
| Lower alveolus | 36.2 | 29.5 | 56.0 | 65.6 | 41.7 | 30.0 |
| Upper alveolus | 24.8 | 16.5 | 0[a] | 31.2 | 19.4 | 13.5 |
| Floor mouth | 20.8 | 42.6 | 65.2 | 33.7 | 26.6 | 14.9 |
| Buccal mucosa | 39.4 | 32.1 | 67.4 | 60.6 | 25.3 | 25.0 |
| Hard palate | 35.3 | 30.4 | 100 | 49.6 | 23.3 | 7.0 |
| Retromolar | 30.6 | 18.0 | 100[a] | 36.7 | 31.3 | 17.6 |
| Oropharynx | | | | | | |
| Base tongue | 16.64 | 16.6 | 89.94 | 35.8 | 21.2 | 4.94 |
| Soft palate | 26.2 | 38.8 | 50.2 | 39.6 | 27.9 | 8.77 |
| Tonsil | 14.95 | 12.1 | 64.1 | 30.8 | 14.37 | 8.3 |
| Oropharynx NOS | 15.3 | 15.7 | 0[a] | 50.8 | 10.3 | 8.3 |
| Hypopharynx | | | | | | |
| Post-cricoid | 15.5 | 12.7 | 0[a] | 36.4 | 13.5 | 2.1 |
| Pyriform fossa | 19.9 | 17.25 | 0[a] | 44.6 | 21.8 | 10.8 |
| Hypopharynx NOS | 8.6 | 11.3 | 100[a] | 0 | 10.7 | 5.2 |
| Nasopharynx | 21.6 | 23.7 | 100[a] | 35.5 | 22.7 | 21.7 |
| Larynx | | | | | | |
| Vocal cord | 70.45 | 60.36 | 78.9 | 60.5 | 55.6 | 52.0 |
| Supraglottic | 27.2 | 38.0 | 74.0 | 61.7 | 28.7 | 9.1 |

[a]Estimate based on five or less cases.

### RESULTS

The clinical characteristics, TNM staging, treatment summaries and observed survival rates for 1970 oral cancer cases are presented in Table 1. The average age of patients ranged from 49.4 years for anterior tongue cancer to 54.1 years in hard palate cancer. The distribution of TNM staging among eight sites of oral cancers showed that except for lower lip (54.9%) and anterior tongue

© Cancer Research Campaign 1998

cancer (46.2%), for most of the sites, patients with stage I and stage II disease together accounted for between 7.9%, for lower alveolus cancers, and 27.7%, for hard palate cancers.

Management of oral cancer depends largely on the stage of the disease. In our series, surgery alone or radiotherapy alone or their combination remained as the primary treatment. The 'other' treatment category mostly included the combination of chemotherapy with either surgery or radiation or both in the management of oral cancer. The 5-year observed survival rate varied between 20% for the upper alveolus and 43% for lower lip cancers.

The comparable data for pharyngeal and laryngeal cancers are presented in Table 2. In contrast to most of the cancers, a marked predominance of men was seen for all the sites except for post-cricoid cancers, for which a female excess was observed. The average age of the patients varied between 38.2 years for cancer of the nasopharynx and 56.6 years for oropharyngeal cancer. The base of the tongue (818 cases) and pyriform fossa (1000 cases) were the two predominant sites observed in this group. The TNM stage distribution of individual sites indicated that 80–90% of cases presented with stage III or IV malignancies, the only exception being for vocal cord cancers for which the percentage was much lower (30%). In view of this, for most of the patients, radiotherapy was administered as the only treatment. In the case of nasopharyngeal cancer, radiation along with chemotherapy was administered for about 44.8% of the patients and the 5-year observed survival rate for vocal cord cancer was about 62%. For all other sites, the 5-year survival rate ranged between 8%, for hypopharyngeal cancers, and 25%, for both soft palate and supra glottic cancers.

The 5-year observed survival rates according to primary treatment for the eighteen sites of head and neck cancer considered are presented in Table 3. Surgery, when used alone for the treatment of oral cancer, was followed by a 30–58% 5-year survival of patients. The 5-year survival for radiotherapy alone in oral and pharyngeal cancers in our study was in the range of 3–24%. Vocal cord cancer, when treated by radiotherapy alone, showed a 62% 5-year survival rate. The certain combinations of treatment showed good percentage 5-year survival rates for particular sites, namely nasopharynx, lower alveolus and supra glottis.

The 5-year relative survival rates by sex and TNM stage for all the 18 sites of head and neck cancer are presented in Table 4. Women showed better 5-year survival rates for cancers of the floor mouth (M, 20.8%; F, 42.6%), soft palate (M, 26.2%; F, 38.8%) and hypopharynx (M, 8.6%; F, 11.3%) than men. The stage of the disease is known to be an important prognostic factor. With the increase in the stage of disease, there has been a corresponding decrease in survival. Although few patients were observed with

**Table 5** Five-year relative survival rates (%) for the Eurocare study (**A**) and the Tata Memorial Hospital Study (**B**)

| (A) | | | | | (B) | | |
|---|---|---|---|---|---|---|---|
| Eurocare study[a] Study period 1978–85 | | | | | Hospital study Study period 1987–89 | | |
| | | | Country (%) | | | | |
| **Site (ICD)** | **Cases** | **5-year survival** | **Highest** | **Lowest** | **Site (ICD)** | **Cases** | **5-year survival** |
| Tongue (141) | 3299 | 39 | Scotland (46) | Poland (15) | Tongue[b] | 1341 | 24.02 |
| | | | Finland (45) | France (28) | Base tongue | 818 | 16.6 |
| | | | Switzerland (43) | The Netherlands (28) | Anterior tongue | 522 | 35.8 |
| Oral cavity (143–145) | 4382 | 46 | The Netherlands (62) | Estonia (33) | Oral cavity[c] | 1529 | 33.2 |
| | | | Finland (51) | Poland (33) | Upper alveolus | 71 | 21.7 |
| | | | England (48) | Italy (38) | Lower alveolus | 340 | 34.3 |
| | | | | | Floor mouth | 88 | 23.4 |
| | | | | | Buccal mucosa | 728 | 37.3 |
| | | | | | Hard palate | 90 | 34.4 |
| | | | | | Soft palate | 142 | 27.6 |
| | | | | | Retromolar | 69 | 27.0 |
| Oropharynx (146) | 2457 | 33 | The Netherlands (45) | Italy (23) | Oropharynx | 535 | 14.7 |
| | | | Poland (44) | Scotland (23) | Tonsil | 346 | 14.6 |
| | | | England (36) | Estonia (27) | Oropharynx | 189 | 15.4 |
| Nasopharynx (147) | 1078 | 38 | Switzerland (84) | Estonia (16) | Nasopharynx | 125 | 22.2 |
| | | | Italy (55) | Scotland (24) | | | |
| | | | Finland (51) | Poland (28) | | | |
| Hypopharynx (148) | 2199 | 19 | The Netherlands (36) | Poland (7) | Hypopharynx | 1255 | 18.0 |
| | | | Scotland (24) | Finland (8) | Post-cricoid | 171 | 14.3 |
| | | | Estonia (23) | Switzerland (14) | Pyriform fossa | 1000 | 19.6 |
| | | | | | Hypopharynx | 84 | 9.2 |
| Larynx (161) | 10 612 | 57 | The Netherlands (73) | France (47) | Larynx[d] | 757 | 41.8 |
| | | | Scotland (64) | Estonia (50) | Vocal cord | 259 | 68.0 |
| | | | England (63) | Denmark (60) | Supraglottis | 491 | 28.0 |

[a]Source, IARC (1995). [b]Includes tongue NOS (one case). [c]Includes palate NOS (one case). [d]Includes subglottis (seven cases).

© Cancer Research Campaign 1998

stage I cancer, the 5-year relative survival rates were in the range 56–100% for oral cancers (except for the upper alveolus), 50–90% for oropharyngeal cancer, 100% for nasopharyngeal cancer and 74–79% for laryngeal cancer. This indicates the effectiveness of conventional treatment in the early stage of the disease.

### DISCUSSION

This is a retrospective analysis of 5595 head and neck cancer patients treated during the period 1987–89. Some other sites, namely upper lip, nasal sinus, max antrum and salivary glands, were not included mainly because of the small number of cases and/or because non-epithelial cancers were prevalent among these sites. Management of head and neck cancer depends largely on the size of the tumour, nodal status and histological variety. For oral cancer, surgery either alone or in combination with radiation had been the modality of treatment. For oropharyngeal and hypopharyngeal cancers, radiation therapy had been administered, largely because of the advanced stage of the disease at presentation. In the case of nasopharyngeal cancer, chemotherapy had been administered along with radiation therapy in a large percentage of cases. Laser surgery had been performed for a few selected sites of head and neck cancer but was not identified separately in our analyses. The sequence of treatments, when more than one was administered, has not been considered. In addition, no attempt has been made to identify the nature of surgery or level of radiation dose or chemotherapy schedule in evaluating the efficacy of the treatment. Furthermore, statistical comparison has not been made of the efficacy of different treatments from the data presented in Table 3, mainly because of the likehood of selection bias and of patients' reluctance to undergo surgery, which often would have involved reconstruction and a long stay in hospital. In this study, we have not looked at disease-free survival to indicate the efficacy of primary treatment for head and neck cancer, and many of the patients during the course of follow-up may have received treatment for recurrences or for metastatic disease that subsequently prolonged their survival; in which case, primary treatment alone cannot be considered to have cured the disease.

In general, among the patients considered for the study, 25% came from the city of Mumbai, about 45% from the State of Maharashtra and the rest from various states in India. Cancer is not a notifiable disease and vital statistics records for various states in India do not provide the cause of death. Patients' follow-up status was ascertained mainly through postal inquiry. Non-availability of complete addresses for the patients and failure to respond to our postal inquiry are the factors responsible for a significant percentage of loss during the first year of follow-up in our study. However, the assumption that patients lost to follow-up by the end of 3 years were dead is probably reasonable given that the majority had stage III or IV cancers the last time that they were seen.

The results of the present study are compared in Table 5 with those from the Eurocare study (IARC, 1995). In our study, the results are available for subsites of all head and neck cancers (fourth digit ICD), whereas in the Eurocare study some sites are only available by three-digit site (e.g. 141 – tongue) or by combinations of three-digit codes, as in the case of oral cavity (ICD 143–145). To facilitate comparisons, grouped results are also given for the Indian data in Table 5, including here the small number of subsites that were excluded from the main body of the study. Although the 5-year survival rates show distinct variation between the sites and subsites, the results obtained in our study are reasonably similar to those observed in the Eurocare study. The importance of reporting survival by subsites is clearly brought out in the case of tongue, oral cavity and pharynx.

Other studies have been reported in the literature either for all sites of head and neck cancer individually or by groups (Flores et al, 1986; Rice and Spiro, 1989; Steinhart and Leinsassaer, 1992; Cole et al, 1994; Sagar et al, 1994; Mishra et al, 1996; Mohanti et al, 1996; Grau et al, 1997). The direct comparison of our study results with those reported in the literature has to be done with caution, however, especially as the majority of studies report observed rather than relative survival rates.

Head and neck cancer constitute about one-third of all cancer seen at Tata Memorial Hospital, Mumbai. The TNM staging distribution of head and neck cancer in the hospital over the years indicates that a high percentage of cases were seen at an advanced stage of the disease (HCR, 1996). The present study also showed a large percentage of patients with advanced disease at presentation. This, in turn, is reflected in the low survival rates observed for some sites of head and neck cancer in comparison with those seen in European countries.

Many epidemiological studies carried out from high-risk and low-risk populations have indicated the association of tobacco, alcohol, the chewing of betel quid and some dietary items with head and neck cancer. Eventually, head and neck cancer control will best be achieved through primary prevention, although earlier diagnosis should also be an aim.

### ACKNOWLEDGEMENTS

The authors wish to thank the staff of the Hospital Cancer Registry for their help and assistance and Miss Hilda Sequeira for typing the manuscript.

### REFERENCES

Cole DA, Patel PM, Matar JR, Kenady DE and Maruyama Y (1994) Floor of the mouth cancer. *Arch Otolaryngol Head Neck Surg* **120**: 260–263

Flores AD, Dickson RI, Riding K and Coy P (1986) Cancer of nasopharynx in British Columbia. *Am J Clin Oncol* **9**: 281–291

Grau JJ, Cuchi A, Traserra J, Firvida JL, Arias C, Blanch JL and Estape J (1997) Follow-up study in head and neck cancer: cure rate according to tumour location and stage. *Oncol Switzerland, Oncol* **54**: 38–42

HCR (1996) Hospital Cancer Registry. Desai PB, Rao DN, Rao RS and Shroff PD (eds), Annual Reports 1984–94. Tata Memorial Hospital, Mumbai.

IARC (1992) *Cancer Incidence in Five Continents*, Parkin DM, Muir CS, Whelan SL, Gao YT, Ferlay J and Powell J (eds), Scientific Publication no. 120, Lyon.

IARC (1995) *Survival of Cancer Patients in Europe*. Scientific Publication no. 132, International Agency for Research on Cancer: Lyon

ICD (1978) *International Classification of Disease*, 9th revision. WHO: Geneva

Mishra RC, Singh DN and Mishra TK (1996). Post operative radiotherapy in carcinoma of buccal mucosa – a prospective randomized trial. *Eur J Surg Oncol* **22**: 502–504

Mohanti BK, Tandon DA, Bahadur S, Rath GK, Tanwar RK, Lal P and Biswal BM (1996) Results of definitive radiotherapy in T1 and T2 glottic cancer – Institute of Rotary Hospital Experience. *Australia Radiol* **40**: 287–290

RGI (1994) The Registrar General of India, SRS-Based Abridged Life Table 1986–90, Occasional Paper no. 1.

Rice DH and Spiro RH (1989) *Current Concepts in Head & Neck Cancer*, American Cancer Society: USA

Sagar SM, McKenna G and Nolan MC (1994) A clinical audit of glottic cancer in Nova Scotia: a paradigm for effectiveness research. *Clin Oncol* **6**: 14–23

Steinhart H and Leinsassaer D (1992) Treatment and management of squamous cell carcinoma of the floor mouth. *Laryng Rhino Otol*, **71**: 556–560

UICC (1978) *TNM Classification of Malignant Tumours*, Harmer MH (ed.), International Union Against Cancer: Geneva

© Cancer Research Campaign 1998


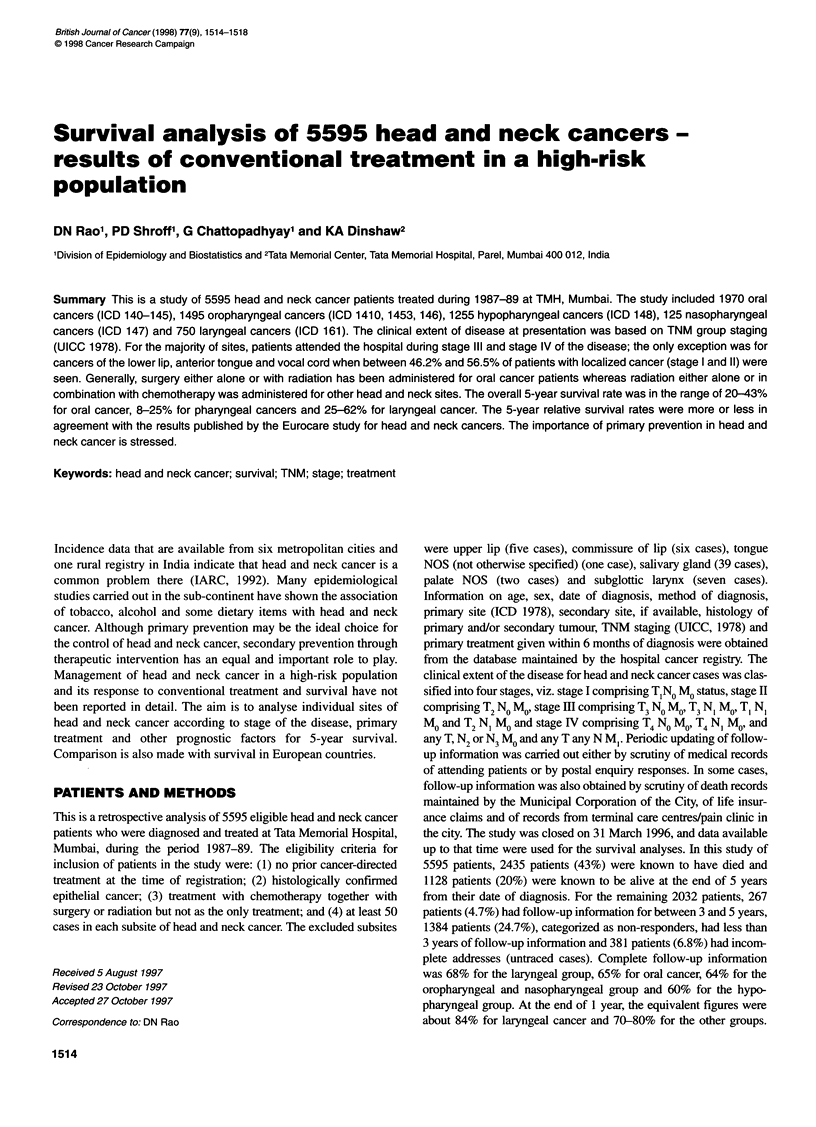

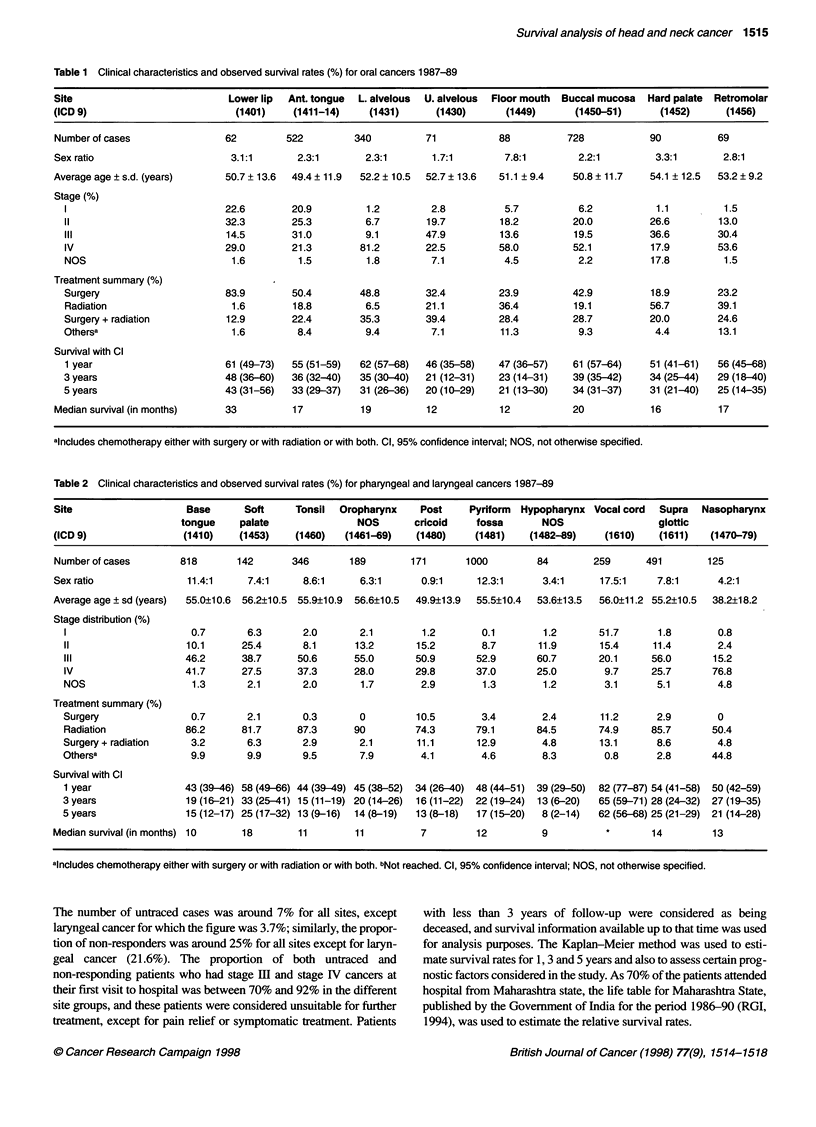

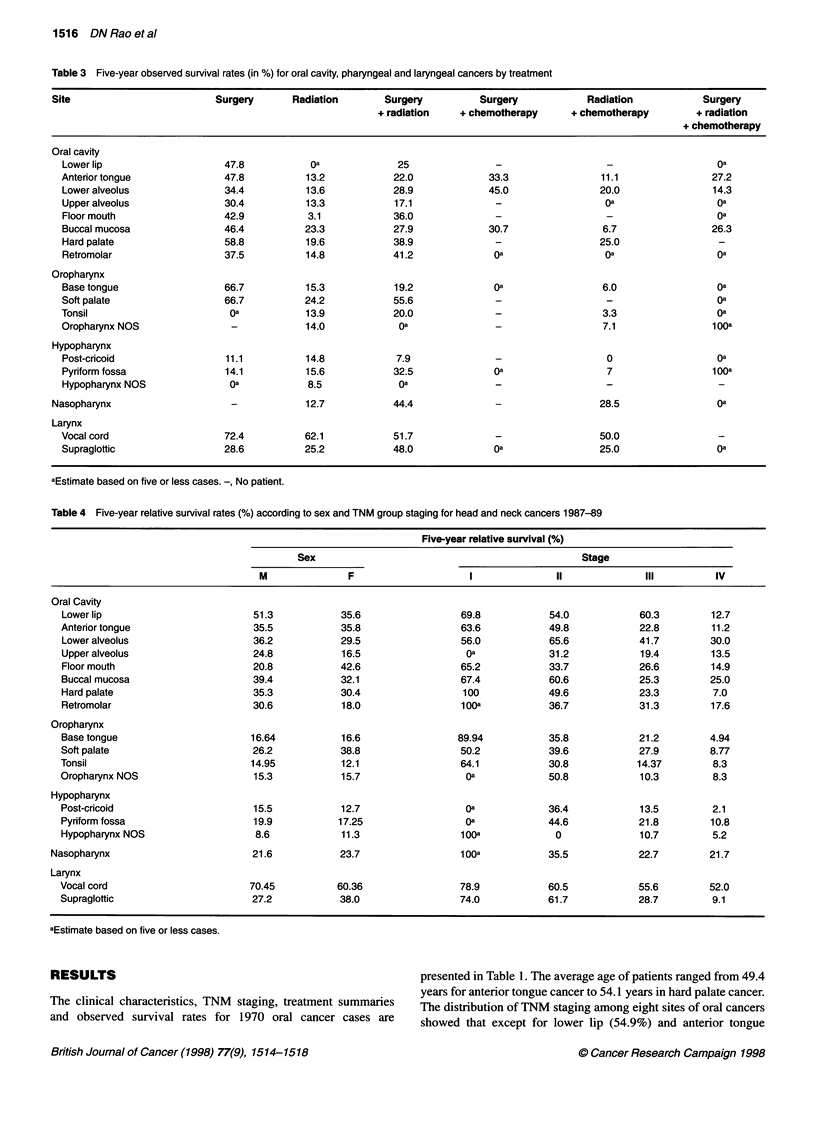

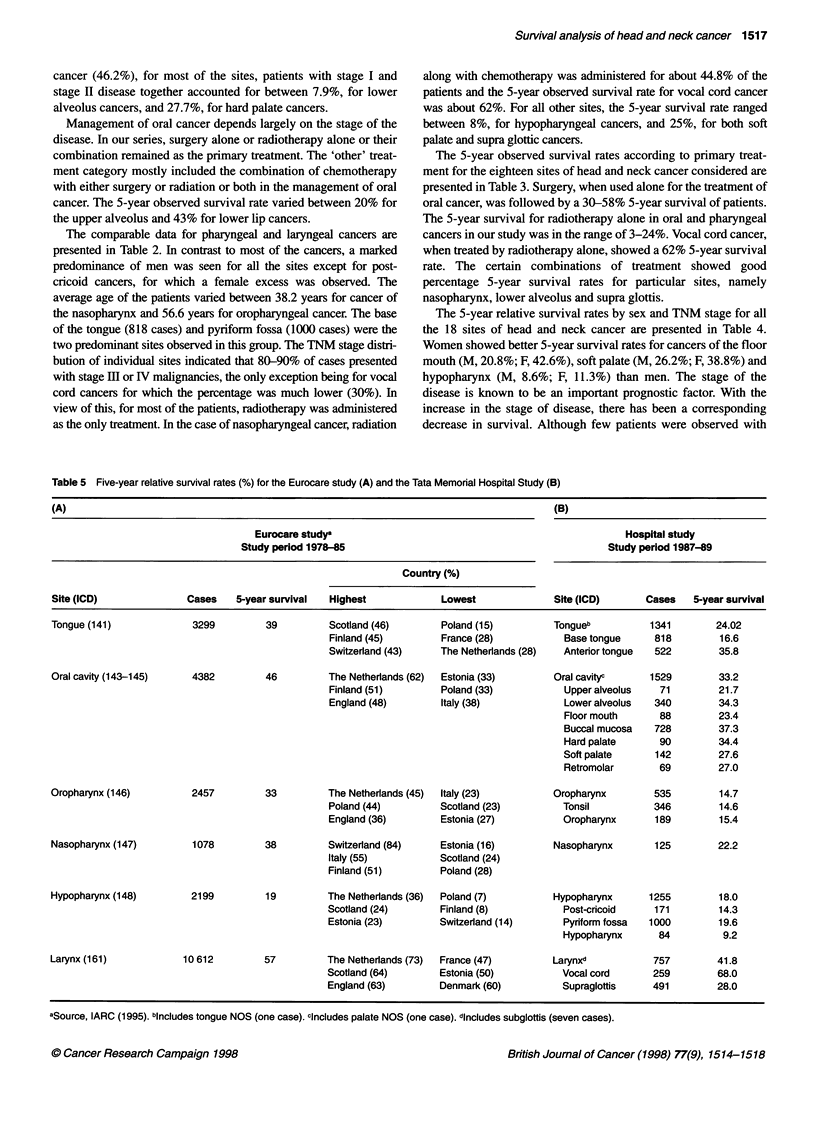

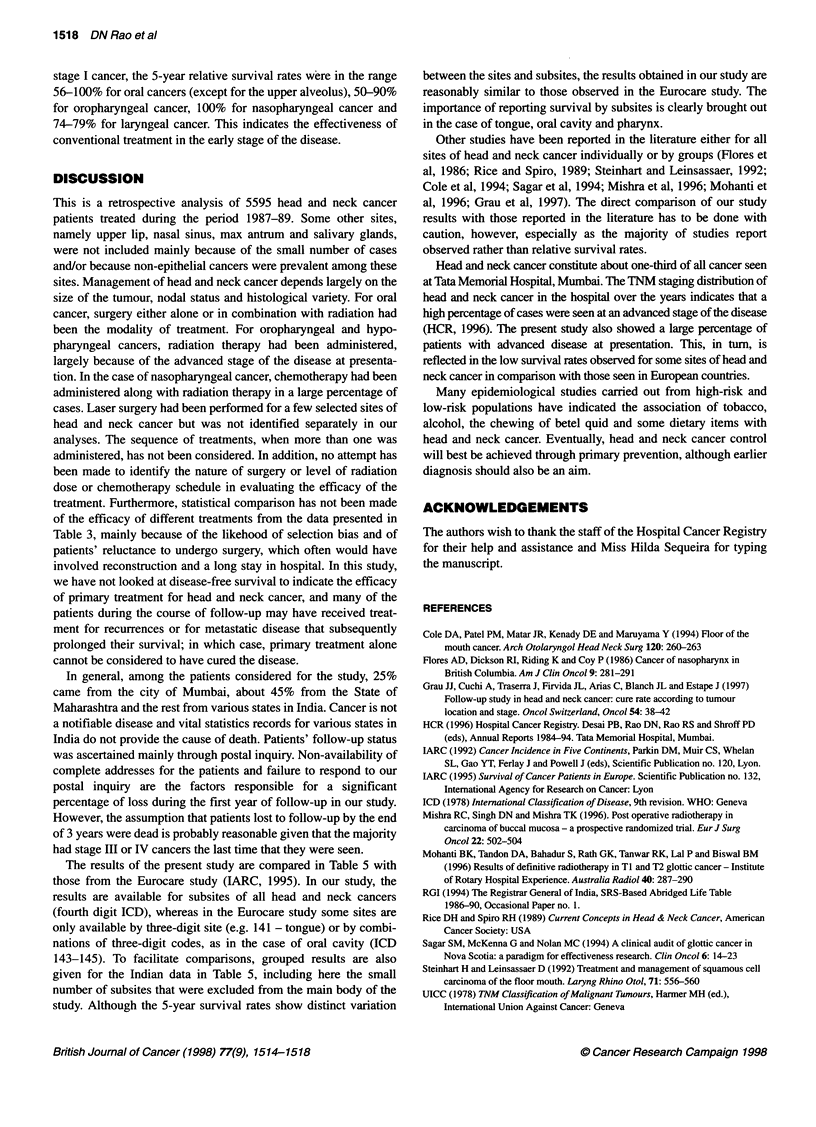


## References

[OCR_00484] Cole D. A., Patel P. M., Matar J. R., Kenady D. E., Maruyama Y. (1994). Floor of the mouth cancer.. Arch Otolaryngol Head Neck Surg.

[OCR_00488] Flores A. D., Dickson R. I., Riding K., Coy P. (1986). Cancer of the nasopharynx in British Columbia.. Am J Clin Oncol.

[OCR_00492] Grau J. J., Cuchi A., Traserra J., Fírvida J. L., Arias C., Blanch J. L., Estapé J. (1997). Follow-up study in head and neck cancer: cure rate according to tumor location and stage.. Oncology.

[OCR_00509] Mishra R. C., Singh D. N., Mishra T. K. (1996). Post-operative radiotherapy in carcinoma of buccal mucosa, a prospective randomized trial.. Eur J Surg Oncol.

[OCR_00514] Mohanti B. K., Tandon D. A., Bahadur S., Rath G. K., Tanwar R. K., Lal P., Biswal B. M. (1996). Results of definitive radiotherapy in T1 and T2 glottic carcinoma: Institute of Rotary Cancer Hospital experience.. Australas Radiol.

